# Electrochemistry of Hypervalent Halogen Compounds

**DOI:** 10.1021/acs.accounts.6c00248

**Published:** 2026-05-19

**Authors:** Igors Sokolovs, Edgars Suna, Robert Francke

**Affiliations:** † Latvian Institute for Organic Synthesis, Aizkraukles 21, Riga LV-1006, Latvia; ‡ Leibniz Institute for Catalysis, Albert-Einstein-Str. 29a, 18059 Rostock, Germany

## Abstract

Hypervalent halogens (I­(III) and Br­(III) derivatives) represent a versatile and effective class of reagents that are widely used in synthetic organic chemistry. Many of these compounds, however, are unstable and pose risks during handling, which limits their practical application, especially on a larger scale. In this context, the electrochemical *in situ* generation of hypervalent halogen species represents an interesting alternative to conventional methods. Inspired by these ideas, we launched a collaborative “trans-Baltic” research program 10 years ago that is still ongoing. Our efforts focus on developing electrochemical methods for synthesis of these reactive species, their synthetic application and mechanistic elucidation. After initial studies on the electrochemical generation of dialkoxy-λ^3^-iodanes, our activities have expanded to include analogous hypervalent bromine compounds as well as diaryliodonium and bromonium salts. Our work has resulted in a large number of new species, most of which exhibit interesting, useful, and versatile reactivity. Their synthesis, electrochemical properties, and reactivity are the focus of this article.

Our review starts with the discussion of fluorinated dialkoxy-λ^3^-iodanes, which are readily formed via anodic oxidation of iodoarenes in fluorinated alcohols such as HFIP or TFE, the latter acting both as solvent and stabilizing ligands. To date, no nonelectrochemical access to such species has been reported, likely due to their sensitivity toward nucleophiles. Although the λ^3^-iodanes derived from iodoarene conversion in HFIP cannot be isolated, they are effective in various oxidative coupling reactions when generated *in situ*. Incorporating ionic tags enables dual functionality as mediator and supporting electrolyte and allows facile recovery after electrolysis. In contrast, analogous dialkoxy-λ^3^-bromanes could not be synthesized electrochemically. However, singly and doubly chelation-stabilized bromanes can be prepared by anodic oxidation in HFIP. The doubly chelated species is significantly more stable and isolable. The less stable singly chelated form can be converted into a stable [Br–O–Br] dimer, which is suitable both as a precursor for other hypervalent bromine compounds and as a reagent for synthetic applications. The intrinsic Br­(III) reactivity of the doubly chelated bromane is moderate but can be activated either thermally or with TfOH, enabling both ionic and single-electron transfer (SET) reactions. In contrast, the [Br–O–Br] dimer undergoes homolytic cleavage upon heating or near-UV irradiation, enabling radical coupling.

When using noncoordinating solvents such as acetonitrile, anodic oxidation of iodoarenes yields iodonium salts. An acid-free, anion-flexible method was developed, allowing counterion variation via the supporting electrolyte and enabling aryl transfer reactions. Compared to the iodonium species, the bromonium analogues are much more difficult to access and are limited to cyclic species formed from 2,2′-dibromobiphenyls. Together with other newly identified hypervalent species, these compounds offer promising starting points for future research.

## Graphical abstract

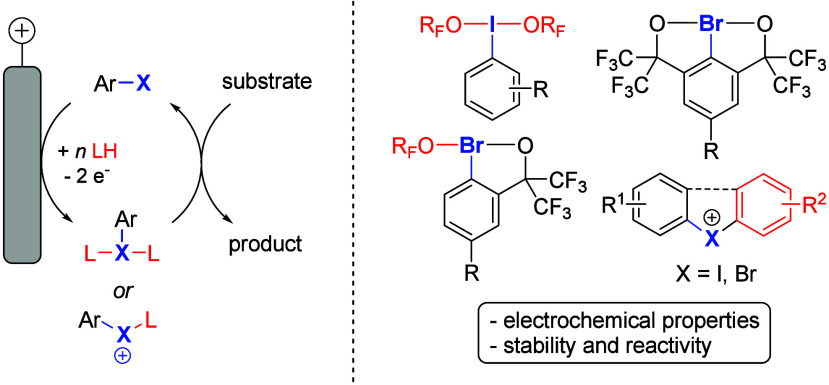

## Key References

-

Koleda, O. ; Broese, T. ; Noetzel, J. ; Roemelt, M. ; Suna, E. ; Francke, R. Synthesis of Benzoxazoles Using Electrochemically Generated Hypervalent Iodine. J. Org. Chem. 2017, 82 (22), 11669–11681 28800234 10.1021/acs.joc.7b01686.^1^ The work describes not only a synthetic application of electrogenerated λ^3^-iodanes but also provides profound insights into the reactivity and stability of the new hypervalent species.
-

Sokolovs, I. ; Mohebbati, N. ; Francke, R. ; Suna, E. Electrochemical Generation of Hypervalent Bromine­(III) Compounds. Angew. Chem. Int. Ed. 2021, 60 (29), 15832–15837 10.1002/anie.202104677PMC836216033894098.^2^ This paper deals with the first electrochemical synthesis of a hypervalent organobromine species and provides first insights into the behavior of these compounds.
-

Mohebbati, N. ; Sokolovs, I. ; Woite, P. ; Lõkov, M. ; Parman, E. ; Ugandi, M. ; Leito, I. ; Roemelt, M. ; Suna, E. ; Francke, R. Electrochemistry and Reactivity of Chelation-stabilized Hypervalent Bromine­(III) Compounds. Chem.—Eur. J. 2022, 28 (42), e202200974 35510557 10.1002/chem.202200974PMC9401590.^3^ This work was devoted to a better understanding of chelation-stabilized λ^3^-bromanes and deals with molecular properties as well as with mechanistic aspects of the electrochemical synthesis and synthetic applications.

## 1 Introduction

Hypervalent iodine compounds represent a versatile and effective class of reagents with numerous synthetic applications, such as carbon–carbon and carbon-heteroatom bond forming reactions,^4,5^ oxidation of functional groups,^6^ halogenation and perfluoroalkylation,^7^ as well as oxidative rearrangements.^8^ In these reactions, stoichiometric (or excess) amounts of hypervalent iodine reagent are routinely used, leading to the formation of aryl iodide byproducts along with the desired compound. Approaches based on the *in situ* generation of hypervalent iodine species from iodoarenes for catalytic applications using terminal oxidants such as *m*CPBA, oxone, or Selectfluor can reduce overall costs, with terminal oxidants remaining a source of stoichiometric waste.^9^ A promising solution to this issue is the use of electricity instead of terminal oxidants to generate the reactive hypervalent iodine species in a reliable and controlled fashion.

Established protocols for the electrochemical synthesis of iodine­(III) compounds are based exclusively on the anodic two-electron oxidation of iodoarenes **1** (Figure 1).^10^ The structure of the resulting iodine­(III) species is governed by both the composition of the electrolyte and the substitution of the aromatic ring. In the presence of (pro)­nucleophiles (Nu^–^/NuH), for example, the formation of λ^3^-iodanes of the type ArI­(Nu)_2_ (**2**) is commonly observed. It is important to note that the scope of suitable nucleophiles is largely restricted to anions such as fluorides, acetates, and fluorinated alcoholates reflecting the limited stability of the corresponding λ^3^-iodanes, and lower oxidation potentials of most nucleophiles relative to iodoarenes.

**Figure 1:**
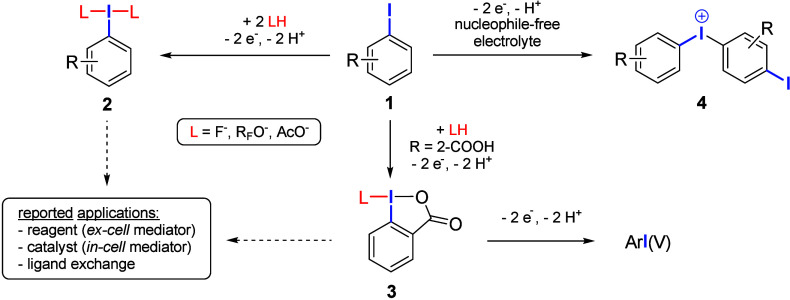
General pathways for the anodic conversion of iodoarenes to hypervalent iodine­(III) and iodine­(V) compounds.

Coordinating *ortho* substituents (e.g., carboxyl groups) typically promote the formation of cyclic structures, e.g., benziodoxoles **3**,^11,12^ which can be further converted to iodine­(V) species under suitable conditions.^13,14^ In the absence of nucleophiles, electro-generated iodine­(III) is stabilized by homocoupling via electrophilic aromatic substitution at **1**, resulting in diaryl iodonium species **4**.^15,16^ In principle, compounds **2** and **3** can serve directly as reagents for the synthetic applications described above, while electro-generated **4** can be employed as an arylation reagent using the electrolyte solution as reactive medium.^17^ Over the past ten years, we have contributed to both areas, beginning with the first work on the electrochemical synthesis of dialkoxy-λ^3^-iodanes in 2016. Our entire work on λ^3^-iodanes and diaryliodonium compounds is summarized in Sections 2 and 3, respectively. Comprehensive reviews on this topic are provided elsewhere.^18–22^

Compared to hypervalent iodine­(III) compounds, the isoelectronic bromine­(III) analogues have been scarcely explored. This deficit can be ascribed to both the difficulties in controlling the reactivity of λ^3^-bromanes and to the challenging synthesis, which usually starts from BrF_3_, a highly toxic and corrosive liquid that requires specific equipment for safe handling.^23–27^ The electrochemical synthesis of λ^3^-bromanes from nonhazardous starting materials thus represents an attractive alternative, yet it has been little investigated to date.^28^ Our activities in this area began around six years ago, documented with the publication of the first electrochemical λ^3^-bromane synthesis in 2021.^2^ A summary of our contributions to hypervalent bromine chemistry is provided in Sections 3 and 4.

## 2 Electrogenerated λ^3^-Iodanes

At the outset of our activities, we focused on the electrochemistry of dialkoxy-λ^3^-iodanes, a compound class that had been little investigated to date. Earlier work by Nishiyama et al. demonstrated that anodic oxidation of iodobenzene in 2,2,2-trifluoroethanol (TFE) renders a hypervalent iodine compound,^29,30^ which cannot be isolated but used as an electrochemically generated reagent for various transformations (a so-called “*ex-cell* mediator”, see Figure 2A).^31^ However, our investigations showed that the oxidation of PhI in TFE competes with anodic decomposition of the solvent. In contrast, 1,1,1,3,3,3-hexafluoroisopropanol (HFIP) provides higher anodic stability and was employed in all subsequent studies.^32^ To overcome challenges associated with salt additives and difficult separation of the iodobenzene mediator from the products, the bifunctional mediator-supporting electrolyte species **5a-ClO**_**4**_ (see Figure 2B) was developed. It features a 4-iodophenyl moiety tethered to a quaternary ammonium group, which ensures ionic conductivity and facilitates recovery from the reaction mixture. **5a-ClO**_**4**_ is prepared in three scalable steps from 4-chlorobutyryl chloride and iodobenzene.

**Figure 2:**
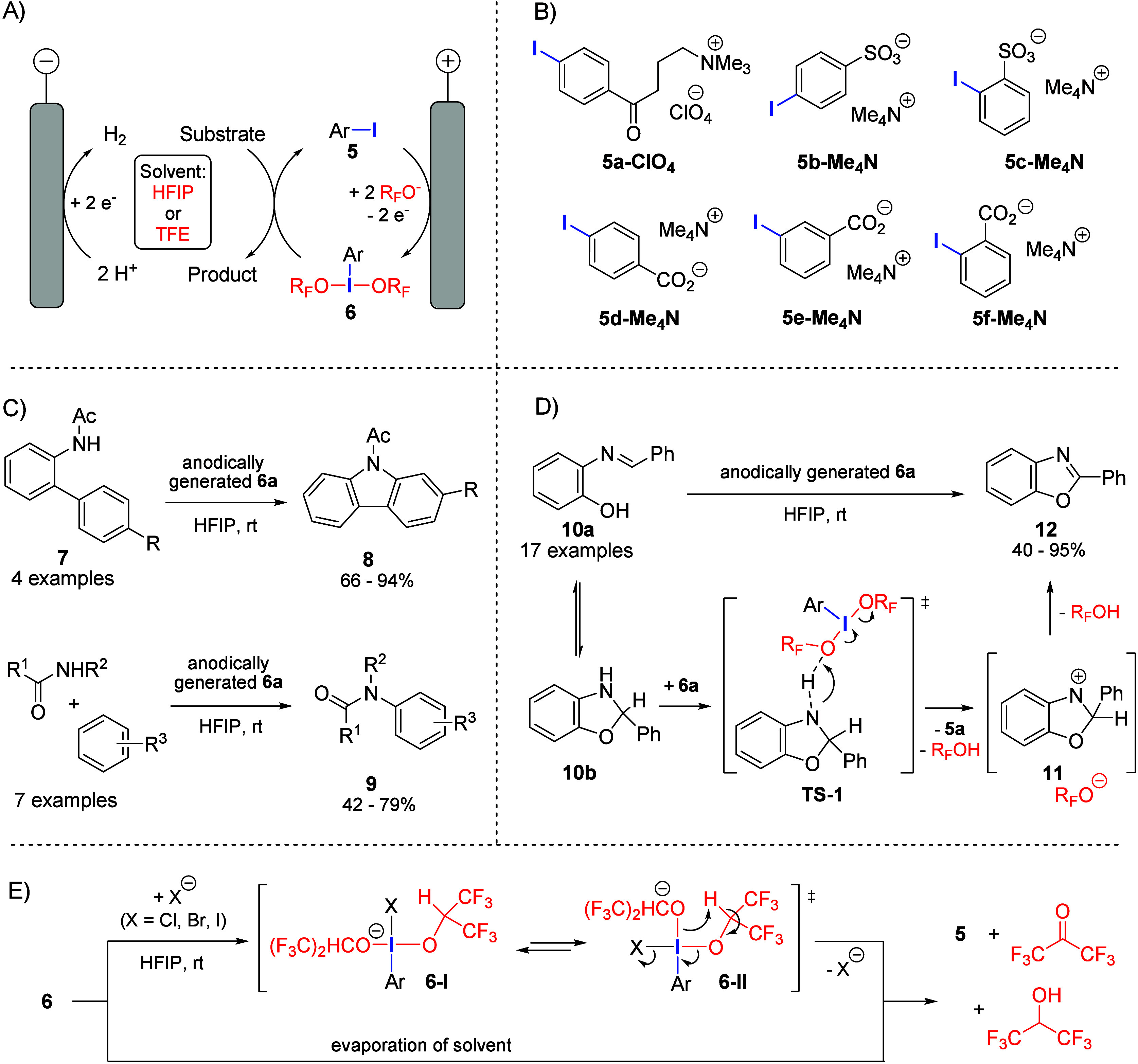
(A) Electrochemical generation of dialkoxy-λ^3^-iodanes **6** from iodoarene precursors **5** for application in iodine­(III)-mediated transformations. (B) Ionically modified iodoarenes **5a–f** serving as supporting electrolyte and *ex-cell* mediator. (C) Applications of **6a-ClO**_**4**_ in inter- and intramolecular amide-arene coupling reactions. (D) Synthesis of benzoxazoles from iminophenols **10** using anodically generated **6a-ClO**_**4**_. Note that **5a-ClO**_**4**_ was used as the mediator in the first two works^1,32^ but was later replaced by **5a-BF**_**4**_ ^33^ for improved safety. (E) Rationale for the lack of stability of dialkoxy-λ^3^-iodanes **6** in the presence of nucleophiles and upon removal of the solvent.

Our synthetic studies have shown that **6a-ClO**_**4**_ induces oxidative intra- and intermolecular C–N coupling between arenes and amide units, enabling the synthesis of substituted carbazoles **8** and amide-arene cross-coupling products **9** in an *ex-cell* mediated process (Figure 2C).^32^ After complete conversion and solvent recovery by distillation, the crude product can be separated from **5a-ClO**_**4**_ by trituration of the solid with a nonpolar solvent. Moreover, it has been demonstrated that **6a-ClO**_**4**_ facilitates the intramolecular cyclization of phenolic imines **10** to benzoxazoles **12** (Figure 2D).^1^ Based on the results of several control experiments and quantum chemical calculations, we proposed a mechanistic sequence which is initiated by equilibrium cyclization to hemiaminal **10b**, followed by reaction with **6a-ClO**_**4**_ in a concerted hydride transfer–reductive elimination via **TS-1**. The resulting nitrenium intermediate **11** is readily deprotonated to give the benzoxazole product **12**.

Further experiments gave useful insights into stability and reactivity of dialkoxy-λ^3^-iodoanes. Analysis of solutions of **6a-ClO**_**4**_ by ^1^H and ^19^F NMR spectroscopy showed that the λ^3^-iodane species is stable in HFIP over several days, whereas the presence of nucleophiles and solvent removal triggers immediate regeneration of **5a-ClO**_**4**_ (Figure 2E). A plausible mechanism involves the addition of the nucleophile to give equilibrating tetra-coordinated isomers **6-I** and **6-II**, ensued by intramolecular β-elimination with concomitant reduction of the iodane unit. Accordingly, the stability of **6** in electrolyte solutions can be attributed to the absence of nucleophiles. This finding rationalizes the failure of nonelectrochemical efforts to synthesize **6a-ClO**_**4**_ (e.g., by oxidation of ArI with peracids or oxone, ligand exchange on ArIX_2_ compounds, etc.)^1^ and underscores the utility of electrosynthesis for the synthesis of novel hypervalent iodine species. A similar β-elimination pathway likely accounts for the reduction of **6a-ClO**_**4**_ to **5a-ClO**_**4**_ upon solvent removal.

Significant improvements in efficiency were achieved by optimizing the structure of the iodoaryl mediator. The electrochemical properties and reactivity of easy-to-synthesize anionic species **5b**–**f** were tested as alternatives to cationic **5a** (Figure 2B).^33^ While the iodobenzoates **5d**–**f** proved impractical for different reasons, iodobenzenesulfonates **5b** and **5c** can be selectively oxidized to the corresponding iodine­(III) species and used for synthetic applications. Using the transformation of **7** to **8** as a test case, carbazole yields between 45% and 88% were achieved after fast and selective generation of hypervalent iodine species **6b-NMe**_**4**_ in a batch process (≥99% *FE* at 100 mA cm^–2^ and 1 F per mole iodoarene). Mediator recovery proved to be straightforward due to the pronounced polarity differences (94–99% recovery rate after trituration of the crude product with a nonpolar solvent). However, in other synthetic applications, the sulfonate tag showed the capability to trap carbocationic intermediates, leading to stable adducts. Consequently, sulfonate mediators **5b-NMe**_**4**_ and **5c-NMe**_**4**_ should be used with caution, with **5a-BF**_**4**_ as the option of choice whenever the interception of carbocation intermediates represents an issue.

## 3 Diaryliodonium and Diarylbromonium Compounds

Over the past decades, diaryliodonium compounds (**13**, Figure 3A) have experienced increasing use as metal-free, easy-to-handle, and efficient aryl transfer reagents.^34,35^ As conventional synthetic methods are often waste-intensive and involve toxic reagents,^36–38^ alternative electrochemical approaches have been developed by other groups to harness the advantages of electricity as traceless and cheap oxidant.^15,16,39,40^ However, these methods typically require strong acids (e.g., H_2_SO_4_ or triflic acid) and afford products with a specific counterion. Since the nature of the counterion can exercise a pronounced influence on the reactivity of the arylation agent,^41–44^ a protocol that enables introduction of the counterion of choice is desirable. In this context, we have developed an acid-free, anion-flexible electrochemical method that provides access to a broad range of structurally diverse (acyclic and cyclic) diaryliodonium salts from both electron-rich and electron-deficient iodoarene/arene precursors (Figure 3A).^17^ The desired anion is introduced via the supporting electrolyte, obviating the need for postsynthesis counterion exchange. Noteworthy, monitoring of the conversion of the iodoarene precursor to **13** can be achieved by ^1^H NMR spectroscopy as well as with a more economic approach based on reverse iodometric titration using ascorbic acid as the reductant.^45^ Scalability of the method was shown by electrolysis on a gram scale. Moreover, successful use of the electrolyte solution as reactive medium in a one-pot procedure was demonstrated using the *O*-arylation of sodium phenolate as a test reaction (Figure 3B), the latter being added to the electrolyte as a THF solution.

**Figure 3:**
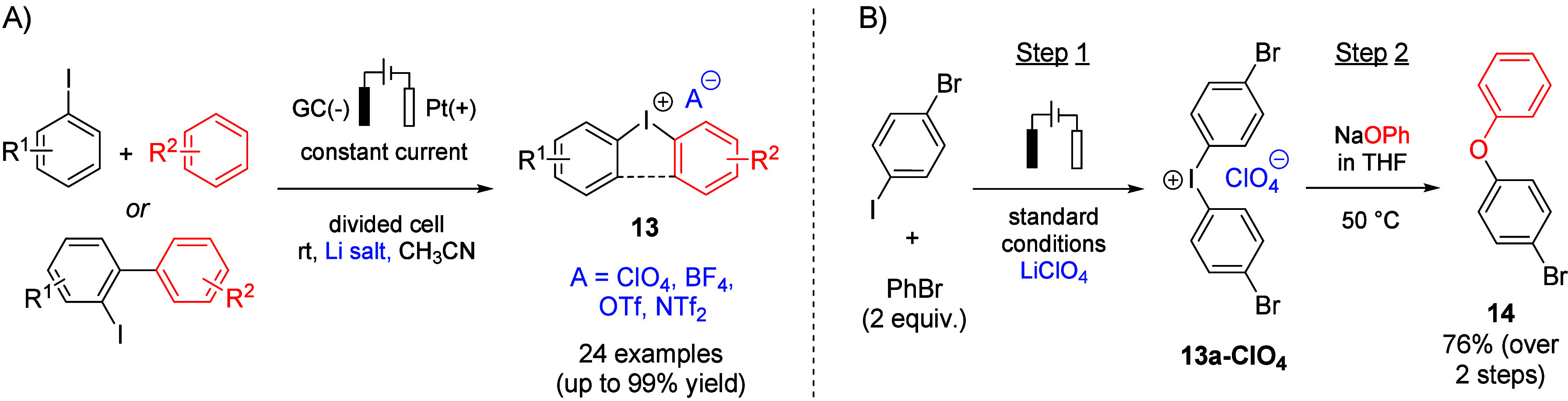
(A) Anion-flexible synthesis of diaryliodonium compounds **13** under galvanostatic conditions. (B) Electrochemical generation of **13a-ClO**_**4**_ and subsequent application as aryl transfer agent using the electrolyte solution as reactive medium.^17^

The solvent acetonitrile used for synthesis of **13** has excellent electrochemical properties but is not considered a sustainable reaction medium. Together with other polar aprotic solvents such as DMF and CH_2_Cl_2_, it is one of the most frequently used solvents in organic electrochemistry. The propylene carbonate-dimethyl carbonate (PC-DMC) system is a promising alternative with enhanced environmental, health, and safety parameters,^46^ and was recently studied by some of us for application in organic electrochemistry.^47^ The systematic study spanned from the characterization of electrolyte properties to evaluation in representative preparative scale reactions. The synthesis of diaryliodonium salts **13** served as an example for a direct (uncatalyzed) anodic reaction, affording yields comparable to those obtained in acetonitrile. An interesting feature is the opportunity for tuning the physicochemical properties of the reaction medium by changing the PC-DMC ratio, thereby modulating the course of electrochemical reactions. This aspect was studied in detail by establishing the relationship between PC-DMC ratio and the yield of diaryl iodonium synthesis as well as the catalytic rate of TEMPO-mediated alcohol oxidations.

The supporting electrolyte LiClO_4_, widely used along with other Li^+^ salts in the study on the synthesis of **13**, is valued for its excellent solubility in polar aprotic solvents, broad electrochemical stability window, and good ionic conductivity. Since perchlorates are strong oxidizers which can even cause explosions when heated with organic compounds, extreme caution is required when using them in the laboratory.^48^ These hazardous properties stand in the way of wider use and scaling up of reactions to the industrial level. Sustainable and affordable alternatives with comparative performance are desirable, which is why we investigated the use of commercially available lithium bis­(oxalato)­borate (LiBOB) as potential replacement.^49^ The study comprises a comparison of the electrochemical properties and the chemical stability to the standard salts, along with a characterization of the performance in three different test reactions. It was found that in principle, LiBOB can compete with LiClO_4_ (and other frequently employed salts such as LiPF_6_ and LiBF_4_) in terms of electrochemical stability and ionic conductivity, but shows increased susceptibility to solvolysis in protic solvents such as methanol. To demonstrate the utility for direct anodic oxidations, 4-bromoiodoarene was converted in acetonitrile in the presence of PhBr and LiBOB, rendering **13a-BOB** in an 84% isolated yield. Noteworthy, the BOB anion is not degraded even after passing excess amounts of charge, as confirmed by ^11^B NMR spectroscopy. Combined with the PC-DMC solvent system, LiBOB thus provides a more sustainable platform for electrosynthesis with potential applicability beyond the studied test reactions.

Diarylbromonium salts display enhanced reactivity as compared to their I­(III) counterparts, which has been attributed to the higher electronegativity and ionization potential of bromine relative to iodine. These species have found broad utility not only as aryl group transfer reagents,^25,50^ but also as effective aryne precursors^51–53^ and Lewis acid catalysts.^54–56^ Despite these advantageous properties, synthetic access to diarylbromonium salts remains limited, relying predominantly on the thermal decomposition of potentially explosive diazonium salts.^52,57,58^ To overcome this limitation, we developed the first electrochemical approach for the generation of cyclic diarylbromonium (III) compounds (Figure 4A).^59^

**Figure 4:**
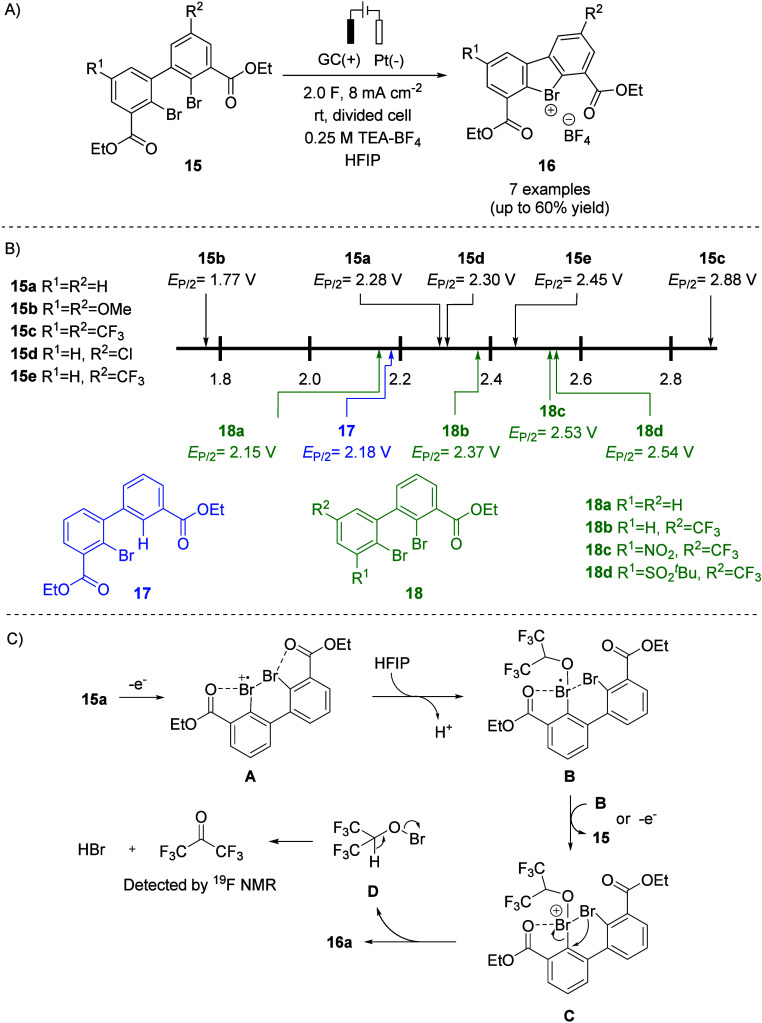
(A) Electrochemical synthesis of cyclic diarylbromonium salts from the corresponding 2,2′-dibromobiphenyls. (B) Half-peak potentials *E*_P/2_ for the oxidation of different 2,2′-dibromo-1,1′-biphenyls measured vs Ag/0.01 M AgNO_3_. (C) Plausible reaction mechanism.

The developed method enables the conversion of 2,2’-dibromobiaryls **15** to the corresponding diarylbromonium salts **16** through an intramolecular cyclization initiated by anodic oxidation. Because one of the two bromine substituents in **15** serves as the leaving group during cyclization, the corresponding monobrominated biaryl **17** undergoes decomposition under the applied reaction conditions and does not afford the desired product. This behavior is in sharp contrast to the reactivity observed for iodo-substituted biaryls (Figure 3A). Moreover, the presence of ester substituents *ortho* to each bromine was found to be essential for the desired reactivity. Both removal of one ester group (**18a**,**b**) and its replacement with an alternative chelating substituent such as NO_2_ (**18c**) or SO_2_^*t*^Bu (**18d**) completely suppresses formation of the desired diarylbromonium salts. Notably, despite these marked differences in reactivity, the measured half-peak potentials for dibromobiaryls that are competent (**15**) or incompetent (**17**, **18**) for cyclization fall within a comparable range (*E*_P/2_ = 1.77 to 2.88 V, Figure 4B). This indicates that the success of anodic cyclization is governed primarily by the substrate structure and not by the redox potentials.

To account for these observations, a possible reaction mechanism was proposed (Figure 4C). The reaction is initiated by single-electron anodic oxidation of **15** to generate the corresponding radical cation **A**. In this intermediate, the Br­(II) center is stabilized through chelation by the adjacent ester group^60^ and the second bromine substituent.^61^ Subsequent coordination of HFIP to the putative Br­(II) center in **A**, followed by deprotonation affords radical intermediate **B**. Disproportionation of **B** or further anodic oxidation then furnishes the corresponding Br­(III) species **C**, which undergoes intramolecular cyclization via an S_N_Ar-type process to deliver biaryl bromonium salt **16**. The hypobromite byproduct **D** formed during cyclization subsequently decomposes to bromide and hexafluoroacetone, the latter of which was detected by ^19^F NMR spectroscopy.^62^ Overall, the developed electrochemical approach to diarylbromonium species represents a safe and inexpensive alternative to the commonly used thermal decomposition of potentially explosive diazonium salts.

## 4 Electrochemistry of Chelation-Stabilized λ^3^-Bromanes

In contrast to monoaryl-λ^3^-iodanes, the corresponding λ^3^-bromanes are considerably more challenging to access via direct two-electron oxidation because of their high oxidation potentials (typically >2 V vs Ag/0.01 M AgNO_3_ in HFIP; see below). In addition, the increased electrophilicity and superior leaving group ability of the bromanyl moiety render monoaryl-λ^3^-bromanes significantly more reactive and, consequently, less chemically stable than their iodine­(III) counterparts. Nevertheless, we have demonstrated that anodic two-electron oxidation of aryl bromides is possible when the characteristic pseudotrigonal bipyramidal geometry of the resulting monoaryl-λ^3^-bromanes is stabilized by coordinating *ortho*-substituents such as the hexafluoro-2-hydroxypropanyl group. Importantly, electrochemical oxidation of aryl bromides **19a**–**g** and **21** to the corresponding monoaryl-λ^3^-bromanes **20a**–**g** and **22**, respectively (Figure 5A), offers significant advantages in safety, sustainability, and scalability compared with the only previously reported method, which relied on chemical oxidation by the highly toxic and hazardous bromine trifluoride.^26,27^

**Figure 5:**
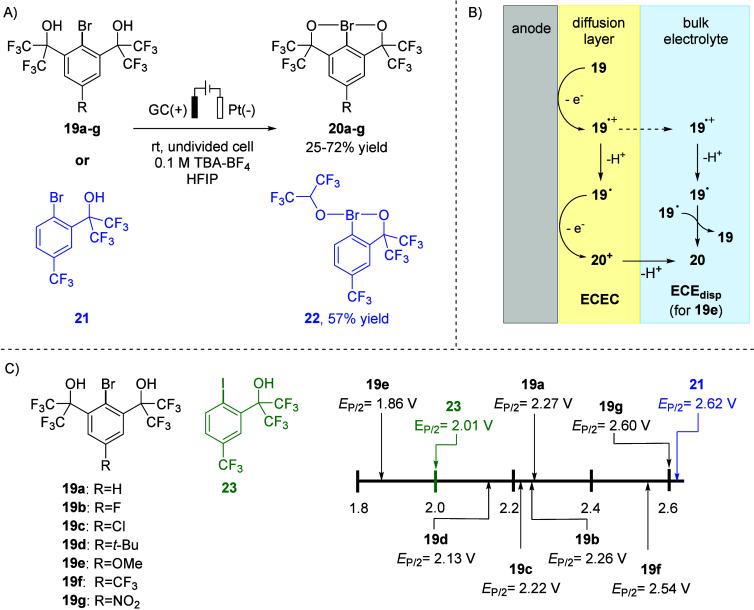
Anodic oxidation of bromoarenes. (A) Synthetic conditions and yields. (B) Mechanism proposed based on voltammetric and spectroelectrochemical experiments. (C) *E*_P/2_ values of bromoarenes 19 and iodoarene **23** measured vs vs Ag/0.01 M AgNO_3_.

The anodic oxidation of aryl bromides **19** and **21** was accomplished in an undivided cell under constant current conditions using a glassy carbon anode. Platinum foil was used as the cathode because of its low overpotential for the proton reduction, which is the desired counter reaction. HFIP proved to be the solvent of choice, whereas MeCN is unsuitable owing to anodic degradation. Double chelation-stabilized λ^3^-bromanes **20a**–**g** (Martin’s bromanes) were obtained in 25–72% yield, whereas benzobromaoxole **22** was formed in 57% yield (Figure 5A). Electrochemical oxidation could be readily conducted on a multigram scale in a single batch.

The *E*_P/2_ values of bromoarenes **19a**–**g** span a range of 0.74 V from 1.86 V for **19e** to 2.60 V for **19g** (Figure 5C). A linear correlation between *E*_P/2_ and σ_*P*_^+^ substituent coefficients for bromoarenes **19a**–**g** was observed, indicating that the oxidation potentials depend on the electronic properties of substituent R. Interestingly, the bromoarene that contains only one chelating substituent (**21**) is harder to oxidize than the *ortho*-disubstituted electron-deficient aryl bromide **19f** (2.62 V vs 2.54 V, both measured in HFIP). As anticipated, the corresponding iodoarene **23** is oxidized at a considerably lower potential (*E*_P/2_ = 2.01 V) than bromoarene **21** (Figure 5C).

Comprehensive electroanalytical and spectroelectrochemical studies, supported by quantum chemical calculations, helped to propose a plausible anodic oxidation mechanism. Accordingly, the λ^3^-bromanes (except for **20e**) are formed via an ECEC pathway involving initial one-electron oxidation, deprotonation by cathodically generated HFIP anions, a second one electron oxidation, and subsequent deprotonation (Figure 5B). In contrast, λ^3^-bromane **20e** is likely generated through an ECE_disp_ mechanism, in which one-electron oxidation of the electron-rich bromoarene **19e**-affords a relatively long-lived radical cation **19e** ^**•+**^ that diffuses into the bulk solution, where slow deprotonation by HFIP-derived alkoxide occurs. Subsequent disproportionation yields **20e** along with regenerated bromoarene **19e** (Figure 5B).^3^

Although benzobromaoxole **22** can be isolated as a crystalline solid and it exhibits moderate stability in organic solvents at room temperature (τ_1/2_ = 96 h in CHCl_3_-*d* and 168 h in MeCN-*d*_3_), the requirement for low temperature (−18 °C) storage limits its practical utility. In contrast, treatment of the **22**-containing electrolysis mixture with TFA followed by aqueous workup afforded the bench-stable dimeric λ^3^-bromane **24** (Figure 6), which can be stored at 4 °C for at least four months without detectable decomposition. The enhanced stability of crystalline **24** relative to **22** renders dimer **24** the most practical product of the anodic oxidation.^63^

**Figure 6:**
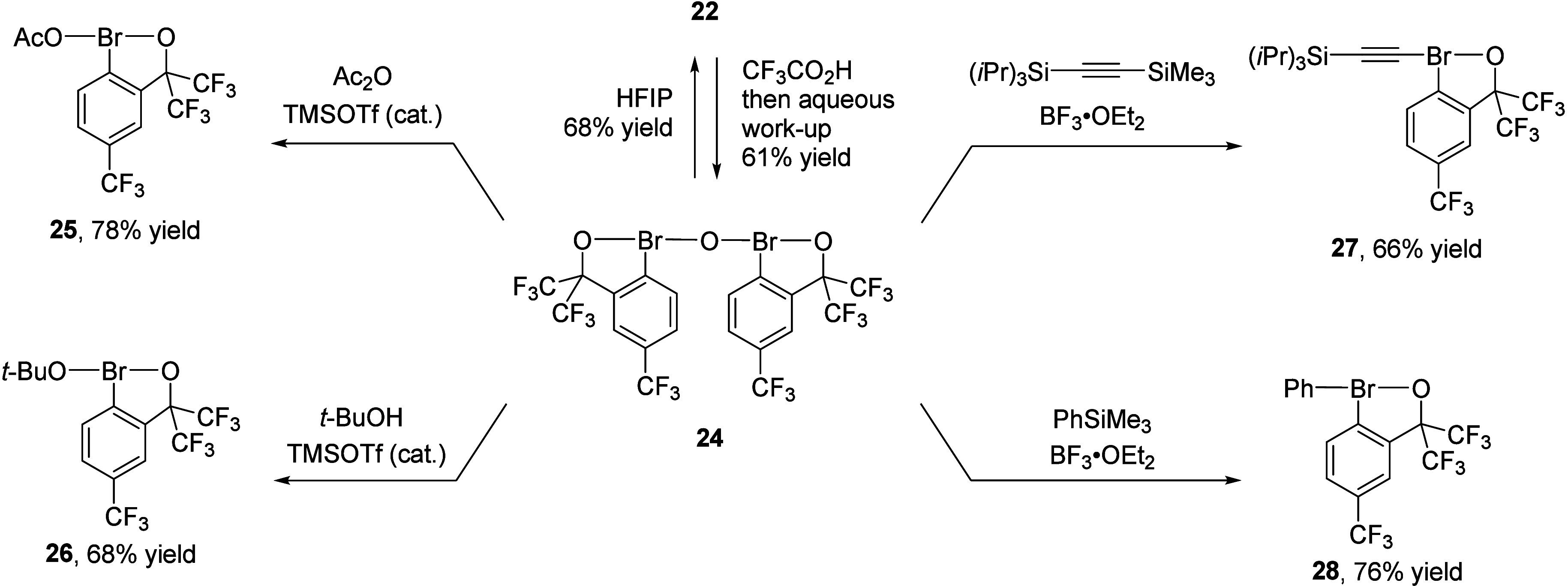
Synthesis of λ^3^-bromanes from **24**.

Easy-to-handle, stable, and crystalline dimeric λ^3^-bromane **24** serves as a versatile precursor for the synthesis of diverse hypervalent bromine­(III) species **22** and **25**–**28** (Figure 6). Thus, treatment of **24** with catalytic TMS-OTf (10 mol %) and acetic anhydride or *tert*-BuOH afforded acetoxy-λ^3^-bromane **25** or alkoxy-λ^3^-bromane **26**, respectively. Activation with stoichiometric BF_3_·OEt_2_ enabled reactions with alkynylsilane and phenyl trimethylsilane to render alkynyl-λ^3^-bromane **27** and diaryl-λ^3^-bromane **28**, respectively. Finally, dimer **24** can be readily converted back to alkoxy-λ^3^-bromane **22** by simple dissolution in HFIP (Figure 6).

The electrochemical properties of the synthesized λ^3^-bromanes were investigated to elucidate their behavior as chemical oxidants. Among the series examined, Martin’s bromanes **20f,g** and dimeric λ^3^-bromane **24** exhibit the least negative reduction potentials (Figure 7A). Consistent with expectations, dimeric λ^3^-bromane **24** is a significantly stronger SET oxidant than the corresponding dimeric iodane **29**, as evidenced by the more negative half-peak potential of – 0.91 V for the latter. In the meantime, all synthesized λ^3^-bromanes are weaker SET oxidants than widely used hypervalent iodine­(III) species such as PIFA and Koser’s reagent PhI­(OH)­OTs (*E*_P/2_ = – 0.33 V and +0.14 V, respectively).^63^

**Figure 7:**
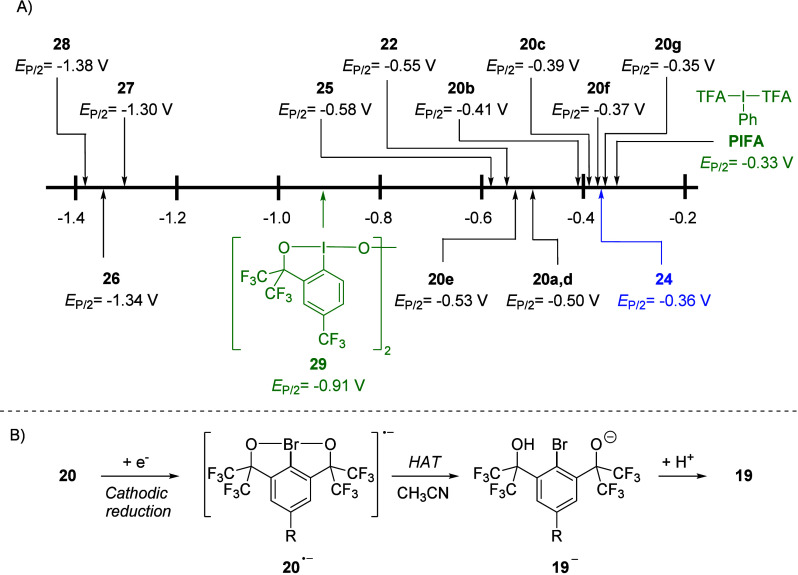
Reduction of monoaryl-λ^3^-bromanes in acetonitrile. (A) Half-peak potentials measured vs Ag/0.01 M AgNO_3_. (B) Mechanism proposed based on cyclic voltammetry and controlled potential electrolysis experiments.

The half-peak potentials of the double-chelation-stabilized Martin’s bromanes **20** span a relatively narrow range of 0.18 V and they correlate well with the electron-donating or withdrawing nature of substituent R (Figure 7A). The reduction potentials of benzobromaoxoles are highly sensitive to electronics of ligands at the Br­(III) center, as illustrated by the remarkable 0.79 V difference between the half-peak potentials of the structurally related HFIP-substituted λ^3^-bromane **22** and *tert*-butoxy-substituted benzobromaoxole **26**. Notably, reduction of λ^3^-bromanes **26**–**28** requires even more negative potentials compared to the dimeric λ^3^-iodane **29** (Figure 7A).

Electroanalytical studies combined with controlled potential electrolysis provided compelling evidence that the cathodic reduction of Martin’s bromanes **20** proceeds via a single electron process involving initial formation of radical anion **20** ^**•–**^, followed by hydrogen atom transfer (HAT) from the solvent to yield **19** ^**–**^.^3^

Despite their comparable reductive half-peak potentials and, consequently, similar single electron transfer (SET) oxidizing abilities, Martin’s reagent **20a** and dimeric λ^3^-bromane **24** exhibit distinct reactivity profiles. Thus, we have demonstrated that Martin’s reagent **20a** promotes oxidative amination of anilines in acetonitrile via an ionic pathway involving intramolecular β-deprotonation within a transient [12-Br-4] complex between the aniline substrate and Lewis acidic λ^3^-bromane **20a** (Figure 8A). In addition, **20a** effects oxidative biaryl coupling of electron-rich arenes, a transformation that likely proceeds through an initial SET oxidation (Figure 8B). This reaction requires activation of the λ^3^-bromane by protonation with TfOH, a strong Brønsted acid that forms *in situ* from the added TMS-OTf.

**Figure 8:**
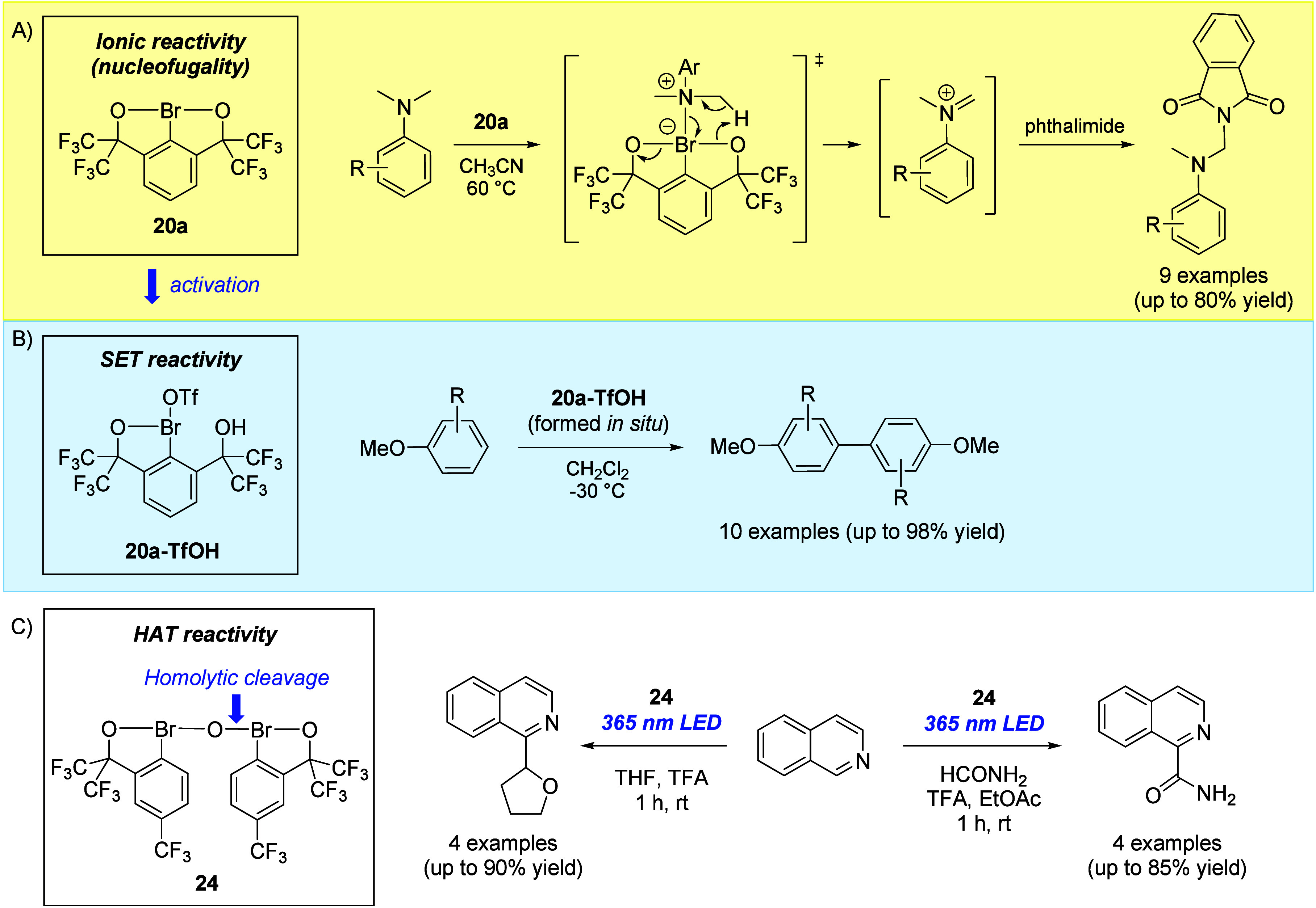
Applications of λ^3^-bromanes **20** and **24**. (A) Oxidative C­(sp^3^)-H amination of aniline. (B) Oxidative homocoupling of anisole. (C) Minisci-type functionalization of heterocycles.

In contrast, dimeric λ^3^-bromane **24** undergoes homolytic cleavage of the exocyclic Br–O bond either thermally (80 °C) or upon irradiation at 365 nm. The pronounced tendency toward Br–O homolytic cleavage renders **24** a convenient source of electrophilic radicals, enabling reactions that are initiated by hydrogen atom transfer (HAT) such as the Minisci-type alkylation of heterocycles with THF and amidation with formamide under photochemical conditions (Figure 8C). Both reactions likely proceed via hydrogen atom abstraction from THF or formamide by the electrophilic oxygen-centered radical, generating nucleophilic carbon-centered radicals that subsequently engage in Minisci-type addition to the protonated heterocycle. Notably, no evidence for Br–O bond homolysis was observed for doubly chelated Martin’s bromane **20f** under either thermal or photoirradiation conditions.

## 5 Conclusion

Over the past ten years, the electrochemistry of hypervalent halogen compounds has been extensively explored by our groups leading to a series of works on λ^3^-iodanes/bromanes as well as diaryliodonium/bromonium compounds. Electrochemical methods have proven highly effective both for accessing novel hypervalent species and for elucidating their properties. In synthetic and mechanistic studies, the electrogenerated species exhibited versatile reactivity, rendering them attractive candidates for various applications. The most important findings of our studies, including similarities and differences between the compound classes examined are summarized below.

Fluorinated dialkoxy-λ^3^-iodanes **6** are readily generated by anodic oxidation of a suitable iodoarene precursor in fluorinated alcohols (i.e., HFIP or TFE), which serve both as solvent and stabilizing ligands. To date, nonelectrochemical access to these compounds does not exist, which we ascribe to the instability of the hypervalent species toward nucleophiles. Although these fluorinated dialkoxy-λ^3^-iodanes cannot be isolated, they are employed as reagents in oxidative coupling reactions when generated *in situ* under electrolysis conditions where the electrolyte also serves as a reactive medium. Incorporation of an ionic tag into the iodoarene scaffold provides a dual benefit, *i*) enabling the iodoarene to act both as redox mediator and supporting electrolyte, and *ii*), facilitating its recovery after completed electrolysis via simple extraction.

Despite intensive efforts, the electrosynthesis of structurally related dialkoxy-λ^3^-bromanes remains elusive. Nevertheless, such species can be obtained by anodic two-electron oxidation of aryl bromides when the alkoxy substituent is tethered to the arene, thereby enabling chelation and stabilization of the Br­(III) center. For example, λ^3^-bromanes **20** and **22** bearing hexafluoro-2-hydroxypropanyl groups are relatively easy to prepare via anodic oxidation in HFIP. The doubly chelated variant **20**, can be easily isolated using column chromatography and conveniently stored. The singly chelation-stabilized version **22** is comparatively less stable. Notably, the electrochemically generated λ^3^-bromane **22** can be readily converted into the more stable [Br–O–Br] dimer **24**, which serves both as a precursor to additional λ^3^-bromanes and as a useful reagent for synthetic applications.

Double chelation effectively attenuates the intrinsic reactivity of bromine­(III) species, but it can be unlocked for synthetic applications either by increasing the temperature or by activation with TfOH. In the presence of such an activator, doubly chelation-stabilized λ^3^-bromanes **20** show both ionic reactivity and pathways consistent with single electron transfer processes. Notably, the [Br–O–Br] dimer **24** displays a distinct reactivity profile, undergoing homolytic bond cleavage upon heating or irradiation with near UV light, thereby providing access radical coupling manifolds.

In noncoordinating solvents (e.g., in CH_3_CN or PC-DMC), the anodic oxidation of iodoarenes leads to the corresponding diaryliodonium salts **13**. A first acid-free and “anion-flexible” electrochemical route was developed, allowing for the introduction of various counterions through the selection of the appropriate supporting electrolyte. The postelectrolysis solution can then be used as a reactive medium for aryl transfer reactions. Once again, the synthesis of structurally analogous bromonium compounds proved to be significantly more challenging. To date, successful synthesis has been achieved under two conditions. First, only cyclic bromonium species are accessible via anodic intramolecular cyclizations of 2,2′-dibromobiphenyls. Second, the biphenyls must bear chelating ester groups in *ortho* position to both bromine substituents. Under these constraints, cyclic bromonium species **16** can be obtained. Collectively, these λ^3^-iodanes and λ^3^-bromanes provide a versatile platform for the development of useful synthetic transformations.
